# l-*threo*-d-*galacto*-Octitol: a curious non-classical order–disorder polytype with an 88 Å axis

**DOI:** 10.1107/S2052520626004907

**Published:** 2026-05-26

**Authors:** Nina Biedermann, Christoph Standfest, Christian Stanetty, Berthold Stöger, Alexander Virovets, Tobias Wolflehner

**Affiliations:** a Institute of Applied Synthetic Chemistry, TU Wien, Getreidemarkt 9, 1060 Vienna, Austria; b X-Ray Centre, TU Wien, Getreidemarkt 9, 1060 Vienna, Austria; c Institute of Inorganic and Analytical Chemistry, Goethe Universitaet Frankfurt, Max-von-Laue-Str. 7, 60438 Frankfurt, Germany; d Institute of Chemical Technologies and Analytics, TU Wien, Getreidemarkt 9, 1060 Vienna, Austria; University of Erlangen-Nürnberg, Germany

**Keywords:** pseudo-symmetry, modularity, OD theory, synchrotron radiation, hydrogen bonding

## Abstract

**GalC8** [l-*threo*-d-*galacto*-octitol, systematic name (2*S*,3*R*,4*R*,5*R*,6*R*,7*S*)-octane-1,2,3,4,5,6,7,8-octol] crystallizes with a pseudo-tetragonal lattice featuring a long (∼88 Å) *c* axis. The bulk of the structure possesses *P*4_3_2_1_2 (*Z*′ = 1) pseudo-symmetry, whereby molecules are arranged linearly with eight molecules in a translation period, thus explaining the long *c* axis. According to this pseudo-symmetry, adjacent molecules are alternately related by 2_1_ screw rotations (in 〈100〉 directions) and twofold rotations (in 〈110〉 directions). Due to an asymmetrical hydrogen-bonding network, symmetry is broken in the vicinity of the twofold rotation axes, leading to an overall *P*2_1_ (*Z*′ = 4) space-group symmetry. The lost symmetry is retained as a twin operation. The structure can be classified as a ‘non-classical’ OD polytype, in the sense that there are nonequivalent layer contacts, yet all members of the polytype family are locally equivalent. The main polytype is not of a maximum degree of order (MDO) and can be described as an alternation of fragments of the two MDO polytypes with *P*4_3_ and *P*2_1_2_1_2_1_ symmetry, respectively.

## Abstract 2

The sugar alcohol l-*threo*-d-*galacto*-octitol crystallizes in a structure with *P*4_3_2_1_2 pseudo-symmetry and a highly elongated unit cell (*c* ≈ 88 Å). In distinct layers of the structure, symmetry is broken by an asymmetric hydrogen-bonding network leading to polytypism, which is discussed in the framework of order–disorder theory.

## 1. Introduction

Waste heat management is a key challenge in solving the energy crisis. Whereas thermoelectrics transform heat into electrical energy, phase change materials (PCM) allow for storage and later recuperation of thermal energy. Inagaki & Ishida (2016 ▸), using crystal structure prediction, postulated exceptionally high thermal storage densities of up to 450–500 J g^−1^ for higher-carbon sugar alcohols with even-numbered carbon chain lengths and ideal stereochemical constitution. In particular, the chiral centers should be in 1,3-*anti* configuration, which allows for two kinds of diastereomers, the *manno* and *galacto* type, as exemplified in Scheme 1 for the C8 sugar alcohols (octitols, eight C atoms). The OH groups of the first two adjacent chiral centers are either in *anti* (*manno* type, blue arrow) or in *syn* configuration (*galacto* type, pink arrow). The remainder of the chain is determined by end-to-end 1,3-*anti* configuration. In Scheme 1, green arrows indicate the ideal 1,3-*anti* configuration.
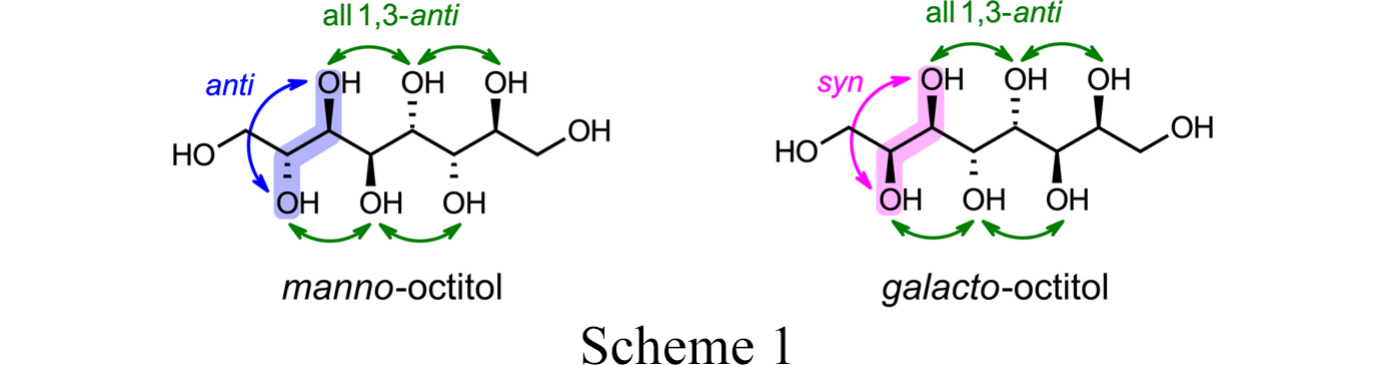


To validate these predictions, we systematically synthesized and conducted structural and partial thermal characterizations of relevant C8 and C10 sugar alcohols of both compound families (Draskovits *et al.*, 2025 ▸; Biedermann *et al.*, 2024 ▸). So far, the experimental data are in close proximity to the predicted values, with a measured thermal storage density of 381 J g^−1^ for *manno*-octitol (Draskovits *et al.*, 2025 ▸), for example. This result is remarkable as this is significantly larger than typical storage densities of organic materials, such as paraffins, fatty acids and high-molecular weight alcohols, which are in the range 150–280 J g^−1^ (Kenisarin, 2014 ▸). Only some natural sugar alcohols with outstandingly high values of up to 350 J g^−1^ (Jadhav *et al.*, 2010 ▸; Tomassetti *et al.*, 2022 ▸) show comparable storage densities. Consequently, it is of great interest to determine the actual crystal structures and relate them to the predicted structures.

The C6 sugar alcohols galactitol (Berman & Rosenstein, 1968 ▸) and mannitol (Berman *et al.*, 1968 ▸; Kim *et al.*, 1968 ▸; Fronczek *et al.*, 2003 ▸) are structurally well characterized. Whereas only one structure of galactitol is known, the polymorphism of mannitol is complex. At least three polymorphs are confirmed (Fronczek *et al.*, 2003 ▸). To our knowledge no structures of the corresponding even-length higher-carbon sugar alcohols have been published. The structure of a single linear C10 sugar alcohol is known (Köll *et al.*, 1992 ▸); however, it features 1,3-*anti* mixed with 1,3-*syn* configuration.

Here we present the structure of the enantiopure C8 *galacto*-type sugar alcohol, which we will hereafter call **GalC8** [systematic name (2*S*,3*R*,4*R*,5*R*,6*R*,7*S*)-octane-1,2,3,4,5,6,7,8-octol]. Due to a large axis and smearing of reflections, data were collected using high-brilliance synchrotron radiation. A pseudo-symmetry analysis to explain the observed structural intricacies will be given. Thereto, we apply a generalization of the order–disorder (OD) theory (Dornberger-Schiff & Grell-Niemann, 1961 ▸), which presents an alternative view of crystalline materials, taking into account the short range of interatomic interactions. Members of an OD family are locally equivalent (and thus energetically very similar), but cannot be superimposed by application of a global isometry of Euclidean space. Instead, they have to be related by partial operations mapping distinct overlapping subspaces, usually pairs of layers.

## 2. Experimental

### 2.1. Synthesis

**GalC8** was synthesized as described in our previous work (Biedermann *et al.*, 2024 ▸) from commercially available l-lyxose via indium-mediated acyloxylation and subsequent dihydroxylation of the obtained enitol followed by Mitsunobu reaction. For crystallization, **GalC8** (10 mg) was suspended in a methanol/H_2_O mixture (4:1, 0.5 ml) in a microwave reaction vial and placed in a Biotage initiator + microwave system. The suspension was heated to 100 °C for 1 min and subsequently allowed to cool down to 70 °C. The vial containing a clear solution was taken out and allowed to slowly cool down to room temperature, leading to crystallization. The colorless crystalline solid was separated via centrifugation, triturated with methanol twice (2 × 1 ml) and pre-dried at 70 °C. Further drying was performed at 40 °C *in vacuo* (<5 mbar) resulting in 8.5 mg of crystalline **GalC8**.

### 2.2. Diffraction

Intensity data of multiple crystals were collected in a dry stream of nitrogen at 170 K on the high-flux P24 beamline of the PETRA III synchrotron facility using 0.560 Å radiation. Two 360° φ-scans with 0.2° frame width were collected. To rule out potential phase transitions, data of a crystal were likewise collected at 300 K. Frames were converted into the ESPERANTO format and processed with *CrysAlisPRO* (Rigaku Oxford Diffraction, 2022 ▸). A correction for absorption effects was performed using the multi-scan approach (ABSPACK). A first solution was obtained from *SHELXT* (Sheldrick, 2015*b* ▸) and the correct symmetry was determined as detailed in *Results and Discussion*[Sec sec3]. The structure was refined against *F*^2^ using *SHELXL* (Sheldrick, 2015*a* ▸) as a twin by pseudo-merohedry. Integration using two separate twin domains resulted in worse refinements, most likely owing to smearing of reflections. Aliphatic H atoms were placed at calculated positions and thereafter refined as riding on the parent atoms. Hydroxyl H atoms were located from difference Fourier maps and refined using distance restraints with respect to the donor and acceptor O atoms. In some cases, the hydrogen positions were not unambiguous and the hydrogen-bonding network could in fact be disordered as detailed below. More details on data collection and structure refinement are compiled in Table 1 ▸.

## 3. Results and discussion

### 3.1. Derivation of the actual and the pseudo-symmetry

Elucidating the actual symmetry of **GalC8** was rather difficult and therefore a detailed account of the encountered challenges will be given. In general, navigating different symmetries (up and down group/subgroup chains), including operations such as origin transformations, was performed using the *JANA2020* suite (Petříček *et al.*, 2023 ▸). Conveniently, *JANA2020* automatically transforms symmetry operations, displacement tensors and refinement restraints. Missed symmetry was checked with the *ADDSYM* routine implemented in *PLATON* (Spek, 2009 ▸).

Intensity measurements using the in-house diffractometer of thin plates of **GalC8** suggested a tetragonal symmetry with a highly elongated unit cell (*a* ≈ 5 Å, *c* ≈ 88 Å). A structure solution with *SHELXT* suggested a *P*4_3_ space-group symmetry with *Z*′ = 2 crystallographically independent molecules. Enantiomorphic space group *P*4_1_ was excluded based on the known absolute configuration of **GalC8**. However, two terminal O atoms were disordered and refinements were of very poor quality (*R*_1_ > 20%). Implementation of the expected twinning by merohedry (twofold rotation about 〈100〉) did not improve the reliability factors.

**Figure 1:**
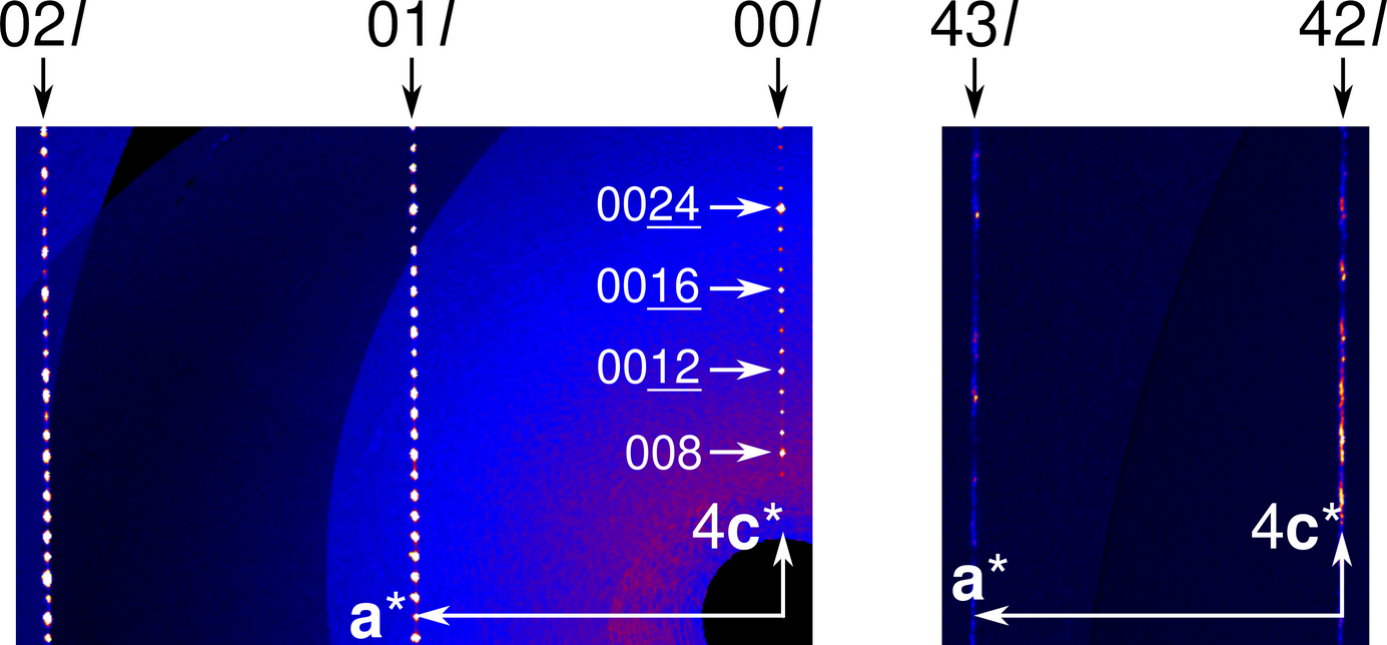
Reciprocal space sections at constant (left) *h* = 0 and (right) *h* = 4 showing well defined reflections close to the origin and more smeared-out/split reflections at larger *h* and *k* values. The reflections expected for a 4_3_ or 4_1_ screw rotation at the (00*l*)* rod are indicated by white arrows. Other reflections on this rod violate the reflection conditions of these operations.

To shed light on the matter, intensity data were collected using synchrotron radiation. While performing these experiments, it became clear that the crystals are most likely not tetragonal as they showed typical signs of pseudo-merohedral twinning. Notably, whereas rods with low *h* and *k* values showed well resolved reflections, at a larger distance from the origin in reciprocal space, reflections were smeared out along the c* direction (Fig. 1 ▸).

Moreover, space group *P*4_3_ could be ruled out owing to violation of the reflection conditions on the (00*l*)* rod (*l* = 4*n*, 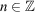) (see Fig. 1 ▸). Regarding the *translationengleiche* (possessing the same translation lattice) subgroups, there are precisely two candidates: *P*4_3_ > *P*112_1_ > *P*1. The intermediate *P*112_1_ can be excluded, as diffusiveness of reflections in c* direction indicates a **c** vector that is not perfectly normal to the (001) plane. Moreover, the (00*l*)* rod does not show the absences expected for a 2_1_ screw rotation in [001] direction (*l* = 2*n*, 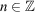). In fact, the disorder did not resolve in *P*112_1_. Therefore, we reduced the symmetry to *P*1 (*Z*′ = 8) and implemented the lost point symmetry as twin operations (fourfold rotation about [001]). This resolved the disorder nearly completely and led to an improved, albeit over-parametrized, refinement, which was evident, for example, in unreasonable displacement parameters.

Applied to the triclinic structure, *ADDSYM* suggested *P*2_1_ symmetry (*Z*′ = 4), *i.e.* a twofold screw rotation axis normal to the (pseudo-)tetragonal axis. Combination of such a 2_1_ axis with the original 4_3_ pseudo-screw rotation gives an *P*4_3_2_1_2 (*Z*′ = 1) pseudo symmetry. Indeed, when manually increasing the symmetry to *P*4_3_2_1_2, a structure with only one disordered non-H atom (a terminal O, see above) was obtained. For unknown reasons, we were unable to obtain this symmetry from *ADDSYM*, even by removal of the atom violating the symmetry. We conclude that reliance on heuristic methods to determine symmetry can be treacherous.

To summarize, **GalC8** crystallizes in a structure with actual *P*2_1_ symmetry that can be derived from an idealized structure with *P*4_3_2_1_2 symmetry in a *translationengleiche* symmetry descent of index 4 (*P*4_3_2_1_2 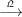
*P*2_1_2_1_2_1_
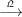
*P*12_1_1 = *P*2_1_). The intermediate orthorhombic phase can be excluded by the arguments given above: it would not produce diffuseness in the c* directions, but rather in the (a*, b*) plane and is in contradiction with the reflections on (00*l*)*.

### 3.2. Crystal structure

**Figure 2:**
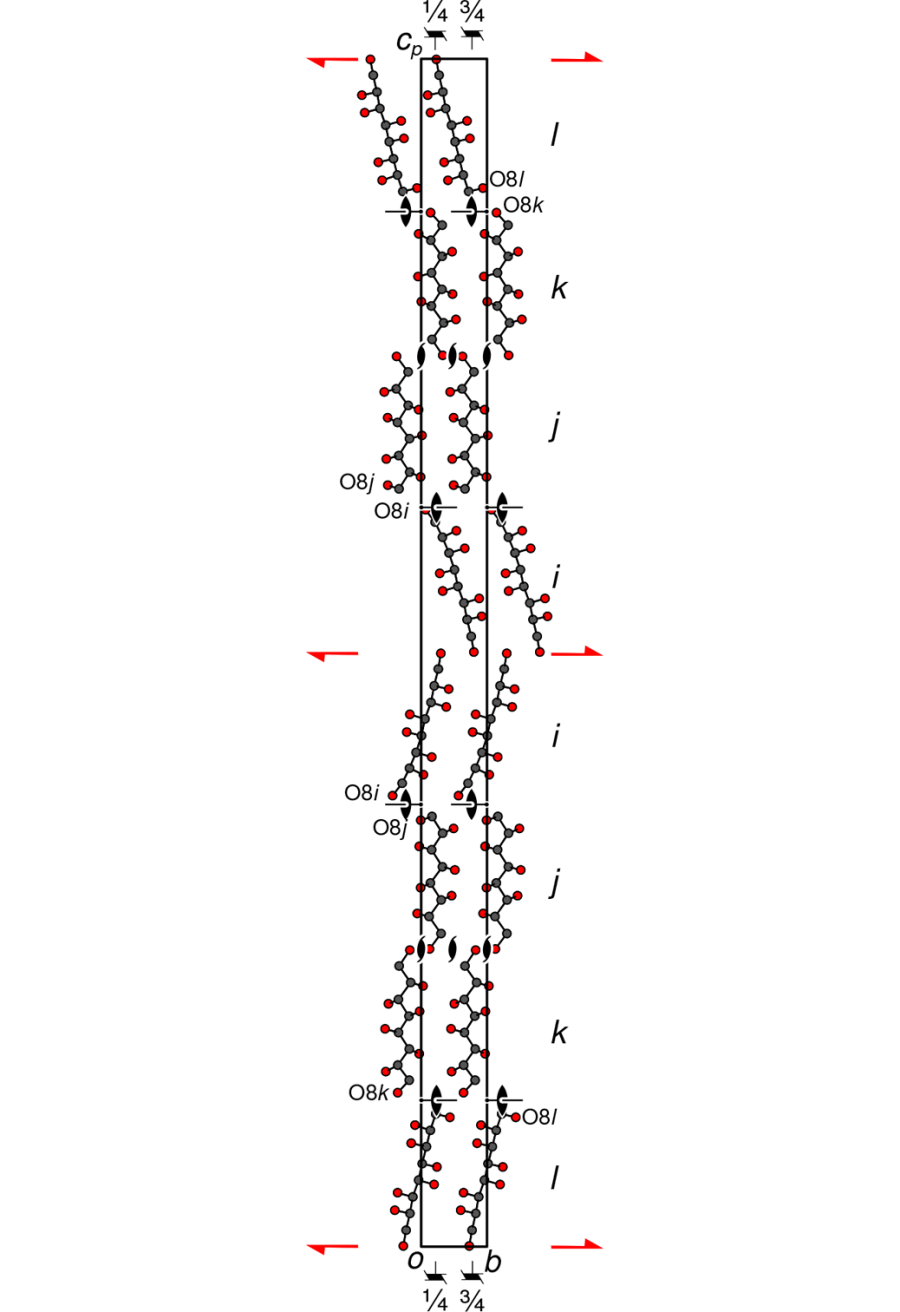
The crystal structure of **GalC8** viewed along [100]. C and O atoms are represented by gray and red spheres of arbitrary radius, respectively; H atoms omitted for clarity. Symmetry elements according to the *P*4_3_2_1_2 pseudo-symmetry are indicated using the standard graphical symbols (Hahn & Aroyo, 2016 ▸). Elements of the actual symmetry are depicted in red. The 4_3_ axes parallel to the drawing plane and the inclined twofold rotation axes are indicated by symbols used for cubic space groups.

Fig. 2 ▸ shows the crystal structure of **GalC8** viewed along [100]. The molecules are arranged linearly along [001]. There are eight molecules in a translation period, related by the (pseudo-)symmetry of space group *P*4_3_2_1_2, indicated by the standard symbols in Fig. 2 ▸. In the [100] and [010] directions, all molecules are related by translation, leading to the highly elongated unit cell.

Adjacent molecules in the [001] direction are related in succession by 2_1_ ([100] direction), twofold rotation ([110]), 2_1_ ([010]), twofold rotation (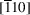), *etc*. Since all these operations invert the orientation of the **GalC8** molecules and the atoms were named linearly (C1 → C8 and O1 → O8), O1 connects to O1 (about 2_1_ screw rotation axis) and O8 to O8 (about a twofold rotation axis).

The only non-H atoms deviating significantly from *P*4_3_2_1_2 symmetry are the O8 atoms (see Fig. 2 ▸). In the idealized *P*4_3_2_1_2 structure, these atoms are disordered in a 1:1 manner about the twofold axis.

**Figure 3:**
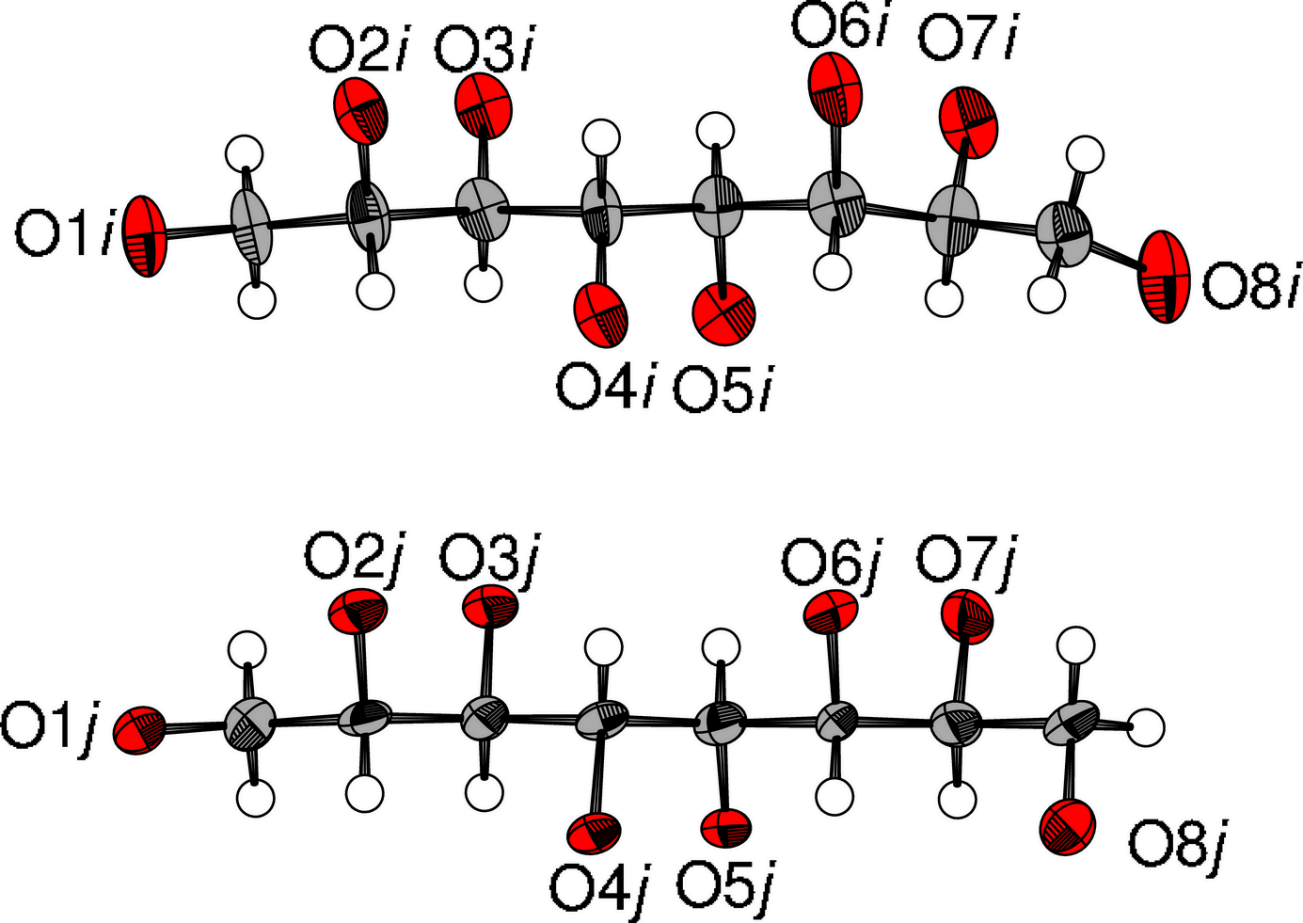
Two distinct conformers of **GalC8** illustrated by molecules (top) *i* and (bottom) *j*. Note the distinct, fully ordered, positions of the O8 atoms. Ellipsoids are drawn at the 75% probability level. In the actual structure, the molecules have opposite orientation with respect to [001] and have been reoriented here for better comparison.

In the actual structure, only the translations and the 2_1_ [010] axes remain (red symbols in Fig. 2 ▸), which means that there are *Z*′ = 4 independent molecules in the asymmetric unit, which are named as letters *i*–*l*. Along [001], the succession is …*lkjiijkl*… (see right-hand side of Fig. 2 ▸). With respect to O8, the molecules appear as pairs of two conformers (Fig. 3 ▸): straight (*i*, *k*) and bent (*j*, *l*), which would overlap in space group *P*4_3_2_1_2. Across the twofold rotation axis one kind of conformer connects to the other via the O8 atoms.

### 3.3. Partial symmetry

The *P*4_3_2_1_2 pseudo-symmetry in **GalC8** applies to parts of the structure but not others. Thus, it is best regarded in terms of *partial* symmetry. Symmetry operations that map only a subset of Euclidean space, such as layers or other modules, are called *partial* (*symmetry*) *operations* (POs). Each PO has a source and a target, which may or may not coincide. The composition of POs forms a space groupoid (Ito & Sadanaga, 1976 ▸). The composition is not closed, since only POs for which the target of the first is the source of the second may be combined. Other than that, the group axioms apply.

An analogous structure, where *P*2_1_/*c* symmetry is only valid for a subset of Euclidean space is the low-temperature phase 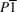 vanillyl oxime (Ehrmann *et al.*, 2019 ▸). In the high-temperature phase, the *P*2_1_/*c* symmetry becomes global. In other words, from a symmetry point of view, the phase transition affects only a subset of the crystal space. In **GalC8** we did not observe a phase transition to a *P*4_3_2_1_2 phase up to 300 K and considering the structural description given below, deem it as highly unlikely.

In OD structures (Dornberger-Schiff & Grell-Niemann, 1961 ▸), owing to partial symmetry, there are multiple ways to connect layers, resulting in equivalent pairs of layers. These pairs can be connected to an infinite number of globally nonequivalent polytypes, which are nevertheless all locally equivalent. Even though **GalC8** is, strictly speaking, not an OD structure, we will apply the techniques of OD theory and show that it nevertheless has OD character.

**Figure 4:**
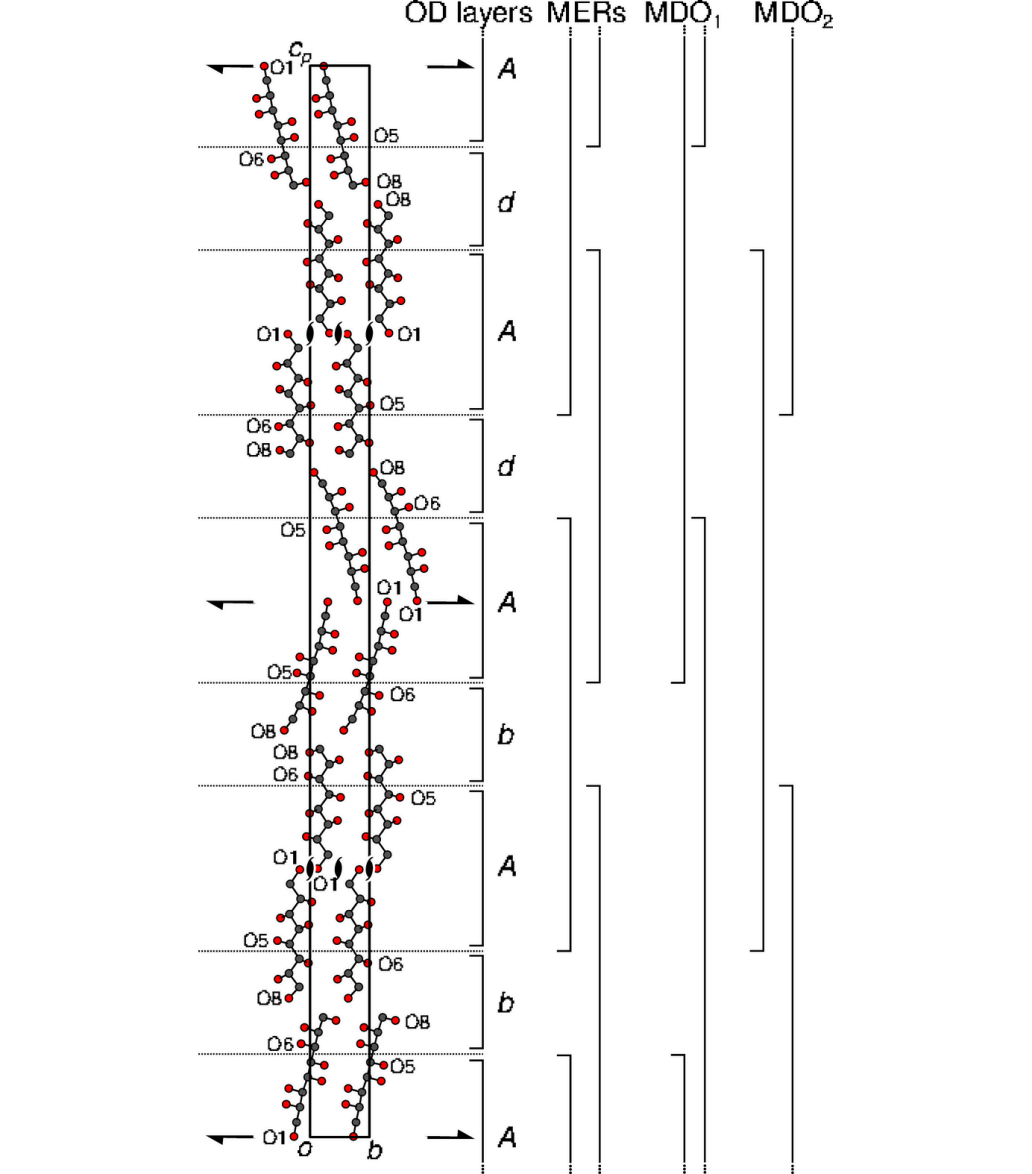
The two kinds (*A* and *d*/*b*) of layers in **GalC8** separated by dotted lines. Atoms as given in Fig. 2 ▸. The (idealized) symmetry elements (2_1_ screw rotations) of the *A* layers are indicated using the standard symbols. Brackets to the right indicate layer *n*-tuples: individual layers, MERs and fragments of the MDO polytypes.

The crucial step in an OD interpretation is a choice of layers (or rods), such that there is only small-to-negligible interactions beyond a layer’s width. As noted in the previous section, the *P*4_3_2_1_2 symmetry applies to all non-H atoms except O8. However, when considering the hydrogen-bonding network (for details see below), different connectivities are also observed for O7 and O6 atoms. Therefore, we will ‘slice’ the molecules at the C5—C6 bond as indicated by dotted lines in Fig. 4 ▸.

From a crystal-chemical point of view it may appear surprising to define layers containing pieces of molecules. Yet, an OD argument is purely based on symmetry and the lack of long-range interactions. For a similar case, see Stöger *et al.* (2013 ▸).

Thus, two kinds of layers are obtained. The O5⋯O1O1⋯O5 layers possess *p*12_1_1 and *p*2_1_11 layer-group symmetry (Kopsky & Litvin, 2006 ▸), respectively (Fig. 4 ▸). In the OD literature, these layers are called nonpolar, because the 2_1_ operation exchanges the layer interfaces, *i.e.* both sides of the layers are related by symmetry. The standard symbol for these kinds of layers is *A* (Dornberger-Schiff & Grell, 1982 ▸).

The O6…O8O8…O6 layers possess only *p*1 layer symmetry, because the 2 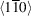 symmetry is broken by the O8 atoms. These layers are called polar, which means that both sides of the layers are not related by layer symmetry and they may, therefore, exist in two orientations with respect to the stacking direction. Depending on this orientation, polar layers are usually designated as *d* and *b* (Dornberger-Schiff & Grell, 1982 ▸) (note that the letters *d* and *b* can be considered as being mirror images of each other).

Thus, **GalC8** is an alternating succession of nonpolar *A* and polar *d* (or *b*) layers. Such a structure is formally not of the OD-kind because it violates the condition that *equivalent sides of equivalent layers contact to adjacent layers such that the resulting pairs are equivalent* (Dornberger-Schiff & Grell-Niemann, 1961 ▸). However, the *A* layer has only one equivalent side that connects to both sides of the *d* layer resulting in the nonequivalent pairs *Ad* and *Ab*.

### 3.4. Equivalent regions and polytypes

The core argument of OD theory is that all structures of an OD family are locally equivalent, because pairs of layers are equivalent. Parts of the structure that are equivalent in the whole family are called equivalent regions (Grell, 1984 ▸). The largest equivalent regions are maximal equivalent regions (MERs). In **GalC8**, given an *A* layer, the adjacent layer can either be *b* or *d*, which means that *A* is located at the boundary of a MER. In contrast, given a *b* layer, to both sides there is only one way of placing the *A* layer. Thus the MERs are *AbA*, or the equivalent *AdA* fragments. The MERs are indicated on the right side of Fig. 4 ▸. In such an MER, the orientations of the two *A* layers to both sides are rotated by 90° about [001], leading to the global tetragonal pseudo-symmetry.

In a classical OD structure, every point in a polytype is covered by two MERs and each MER is at least two layers wide, which means that every point is at least half of a layer inside a MER. It is in that sense that all structures of an OD family are locally equivalent. As can be seen in Fig. 4 ▸, in **GalC8**, some parts (the *d*/*b* layers) are covered by only one MER. However, since the MERs are three layers wide, any point is still at least one half *A* layer inside a MER. Thus, here likewise all members of the structure family are locally equivalent in the same sense as in classical OD structures. We have encountered such a family of structures built of polar and nonpolar layers before [Ca_5_Te_4_O_12_(NO_3_)_2_(H_2_O)_2_, (Stöger & Weil, 2013 ▸)] and named it a non-classical OD family.

All members of the structure family can be constructed by combining MER fragments such that the *A* layers overlap. Owing to the *p*12_1_1 (or *p*2_1_11) symmetry of the *A* layers, there are two ways of extending an *AbA* fragment, as shown in Scheme 2.
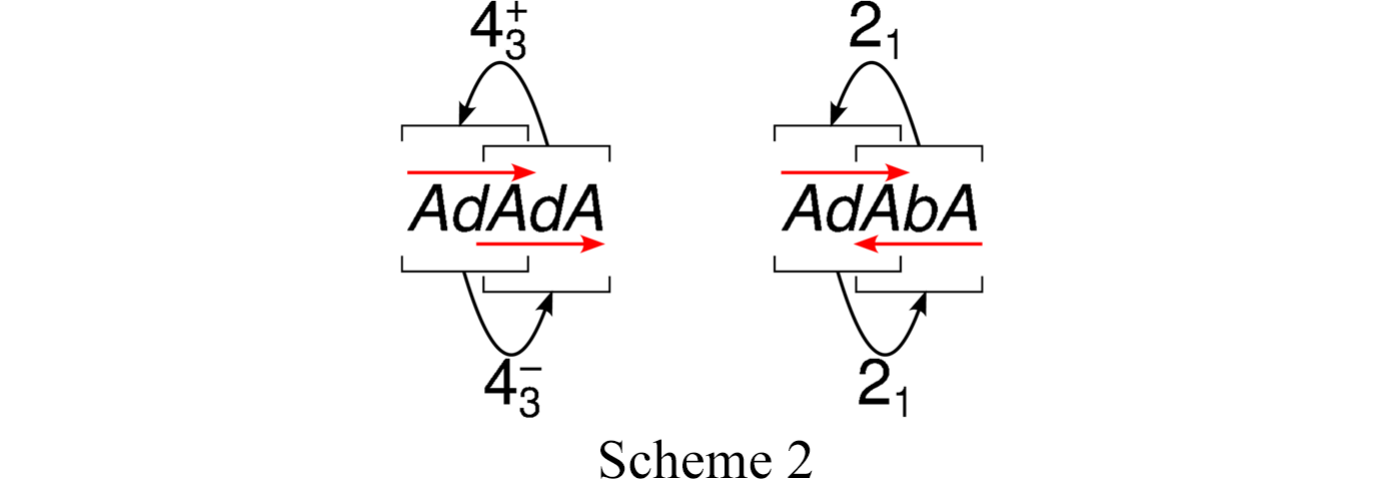
Application of a 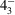 screw rotation generates an *AdAdA* fragment, where both *AdA* fragments possess the same orientation with respect to [001] as indicated by red arrows pointing in the same direction. Here 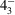 stands for a screw rotation operation with counterclockwise rotation about 90° and intrinsic translation 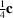. The inverse operation is 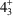: clockwise rotation and translation 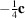. The (layer group) symmetry of the fragment is *p*111, because only the translations of the central *A* layer map the *d* layers onto themselves.

The second way is application of the 2_1_ screw rotation of the third layer of the *AdA* fragment, leading to an *AdAbA* fragment, where the *AdA* and *AbA* MERs possess different orientation with respect to [001], as shown by red arrows pointing in opposite directions. The *AdAbA* fragment possesses *p*2_1_11 or *p*12_1_1 symmetry, because the 2_1_ operation maps *AdA* on *AbA* and vice versa.

Thus by repeated application of 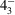 and/or 2_1_ operations, an infinite number of polytypes can be constructed. All of them, if only the *A* layers are considered, possess *P*4_3_2_1_2 symmetry, because the positions of the *A* layers are fixed. Including the *d*/*b* layers, the symmetry is reduced. In fact, the obtained structures need not even be periodic in the [001] direction.

The polytypes that cannot be decomposed into fragments of simpler polytypes are said to be of a maximum degree of order (MDO) (Dornberger-Schiff, 1982 ▸). There are two **GalC8** MDO polytypes:

MDO_1_: *P*4_3_, generated by repeated application of 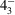 [001], contains only *AdAdA* or only *AbAbA* fragments.

MDO_2_: *P*2_1_2_1_2_1_, generated by repeated application of 2_1_ 〈100〉, contains only *AdAbA* and *AbAdA* fragments.

Note that these were two structure models we considered, but rejected based on the diffraction data (see above), as their symmetries are the maximal *translationengleiche* subgroups of *P*4_3_2_1_2. All other polytypes can be decomposed into five-layer wide fragments of MDO_1_ and MDO_2_.

In particular, the actually observed polytype with *P*2_1_ symmetry corresponds to alternating fragments of MDO_1_ and MDO_2_. This is the simplest possible non-MDO polytype. Two (very small) residual peaks in the difference electron density close to O8*k* and O8*l* indicate sporadical reversal of the *d*/*b* layer at this contact, which suggests the occurrence of other fragments, as is common in structures with OD character.

### 3.5. Hydrogen bonding

Hydrogen bonds of the type O—H⋯O in alcohols are of comparable strength to those in water (Steiner, 2002 ▸). Therefore, the packing of sugar alcohols in the solid state is largely determined by intermolecular hydrogen bonding, which is therefore an essential part of the structure. In **GalC8**, it plays a crucial role in breaking the idealized symmetry.

As noted in *Experimental*[Sec sec2], assignment of the H-atom position was not always unambiguous and the hydrogen-bonding network might be disordered. From the O—O distances, it is however clear between which O atoms the hydrogen atoms are located. Only the donor and acceptor might be reversed.

We will therefore discuss the hydrogen bonding in terms of non-directed graphs, where nodes represent O atoms and vertices represent hydrogen bonds connecting two O atoms. More precisely, we will use voltage graphs (Eon, 2016 ▸), which allow a concise depiction of translationally periodic hydrogen-bonding networks infinitely. All hydrogen bonds in **GalC8** are intermolecular. The donor–acceptor distances are compiled in Table 2 ▸. They all fall into the moderate-strength category according to the classification of Jeffrey (1997 ▸), which corresponds approximately to the hydrogen-bond strength of water.

**Figure 5:**
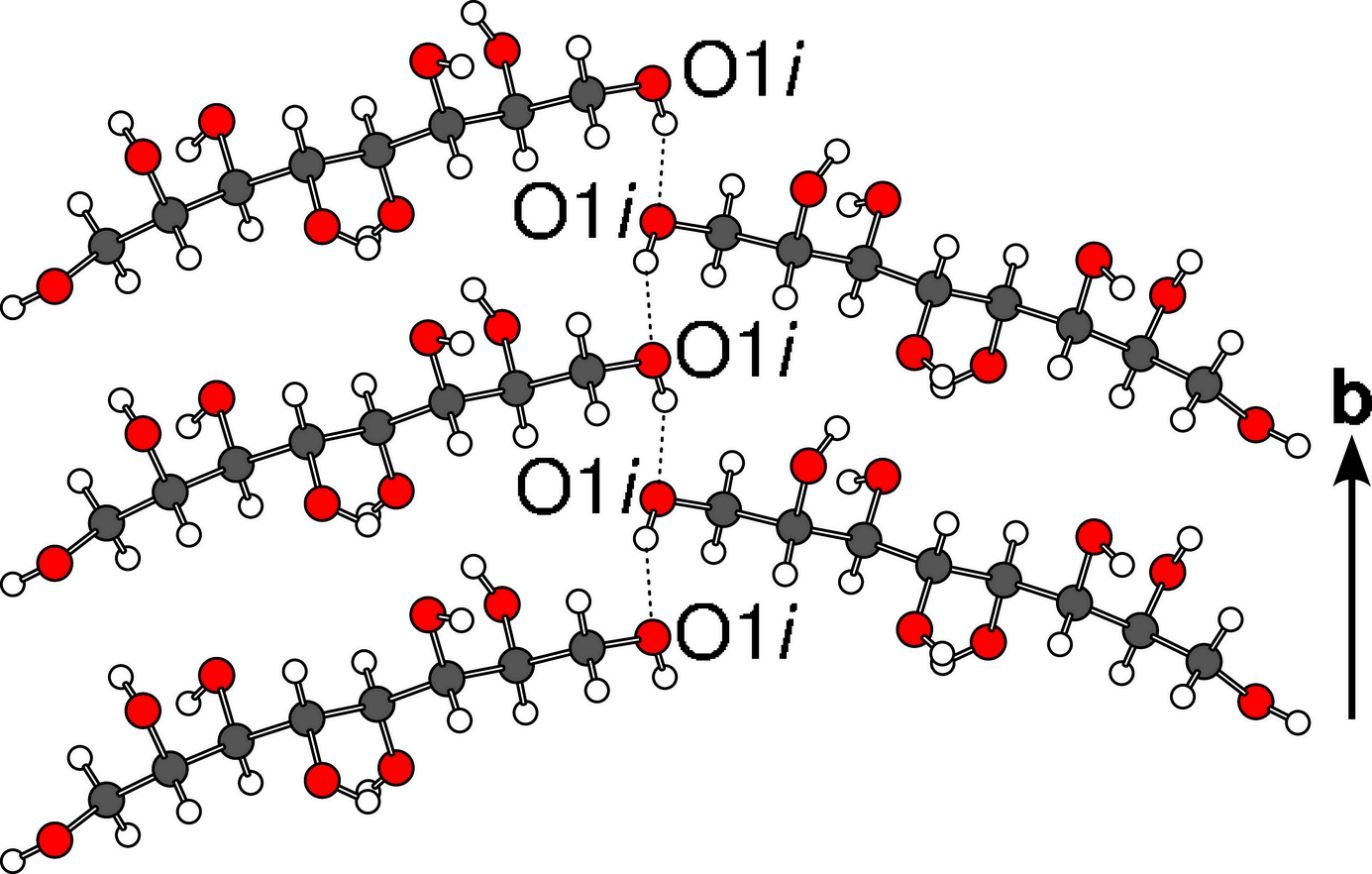
Infinite O1 hydrogen bonding exemplified by the *i* molecule. H atoms are represented by white spheres, other atoms as given in Fig. 2 ▸.

First, let us discuss the hydrogen bonding in the *A* layers. The O1 atoms of two molecules related by the 2_1_ operation form infinite chains in the 〈100〉 directions as shown in Fig. 5 ▸ for the *i* molecules, where the 2_1_ operation of the *A* layer is a global operation of the *P*2_1_ polytype (in the [100] direction the 2_1_ operation is not a global operation).

The corresponding graph is
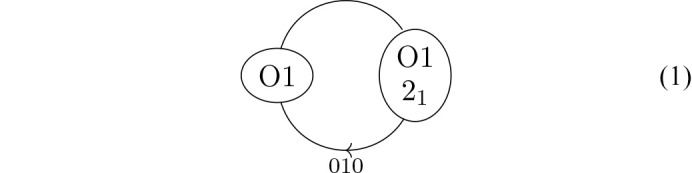
where 2_1_ designates the molecule related by the 2_1_ operation. The label on an edge is called a voltage and implies that when crossing the edge, a translation is performed with respect to the original node. The arrow head in the middle of the edge does not represent the direction of the hydrogen bond, but rather gives meaning to the voltage. Here, crossing in direction of the arrow means translation along [100] and against the arrow along [100]. Note that the voltages are given for one particular orientation of the *A* layers. In half of the *A* layers they are exchanged for 010.

One can also form the quotient graph with respect to all symmetry operations, not only translations (Eon, 2016 ▸), though then the situation may become more complex (McColm, 2024 ▸). Factoring out the (pseudo-)screw rotation, one obtains the graph
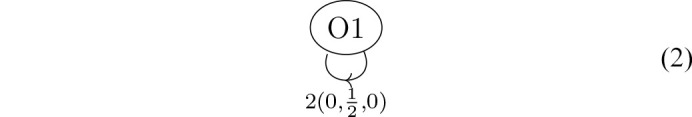
where 2(0, ½, 0) is a twofold screw rotation with intrinsic translation ½**b**. Passing the edge in the same direction adds a translation component of ½**b** per loop and thus the quotient graph indeed represents a periodic hydrogen-bonding network.

**Figure 6:**
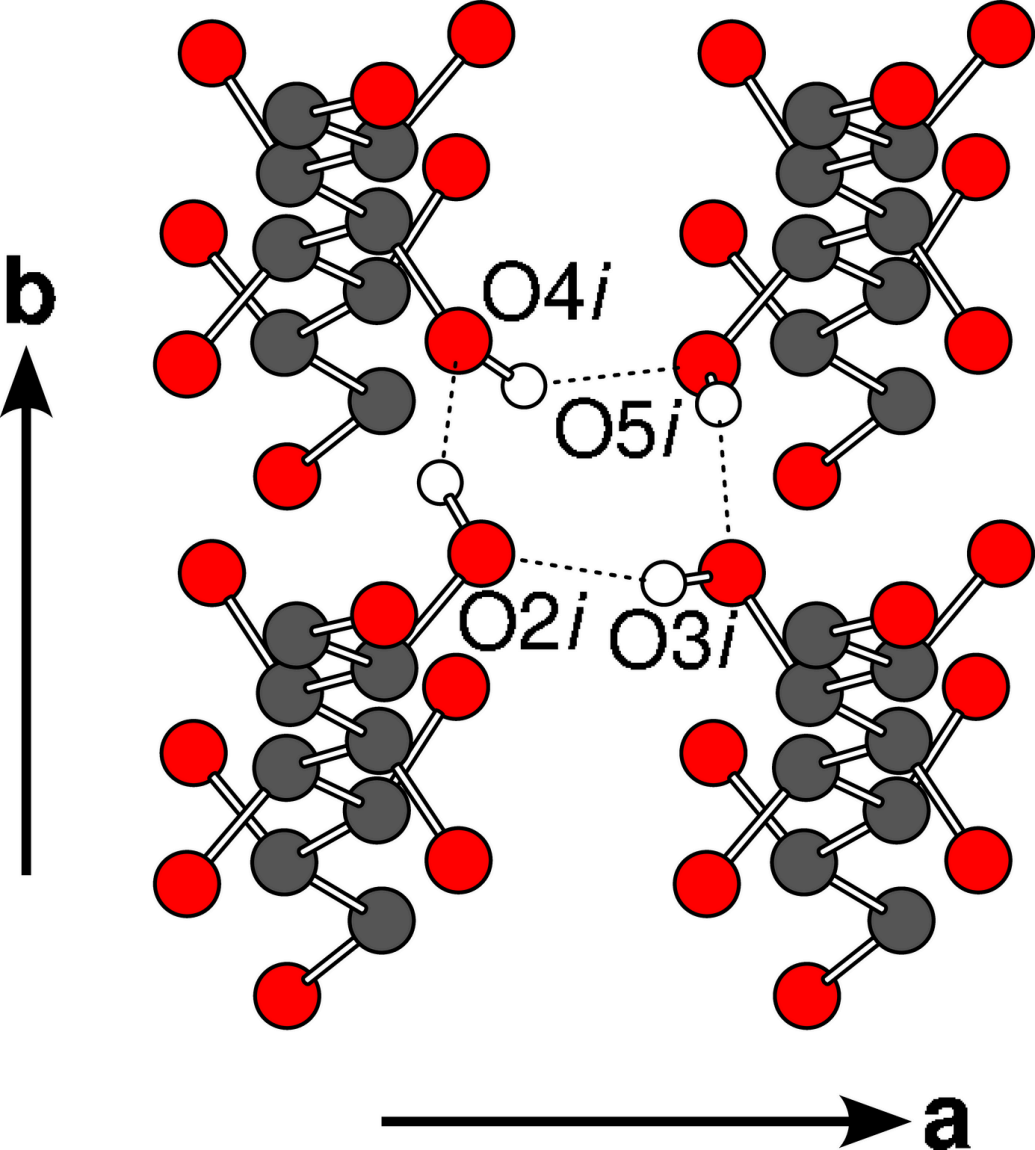
Cyclic O2—O5 hydrogen bonding exemplified by the i molecule, viewed along [001]. Atoms as given in Fig. 5 ▸. H atoms not involved in the cycle have been omitted for clarity.

The O2–O4 atoms are part of a cyclic four-atom network represented by the graph
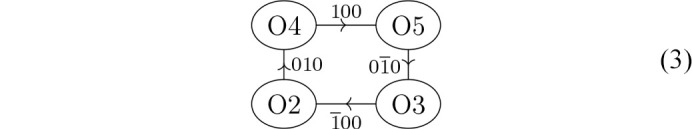
and shown in Fig. 6 ▸. Note that the voltages sum to 000 when taking a full round, which means that the original O atom is reached, as required for a cyclic network.

**Figure 7:**
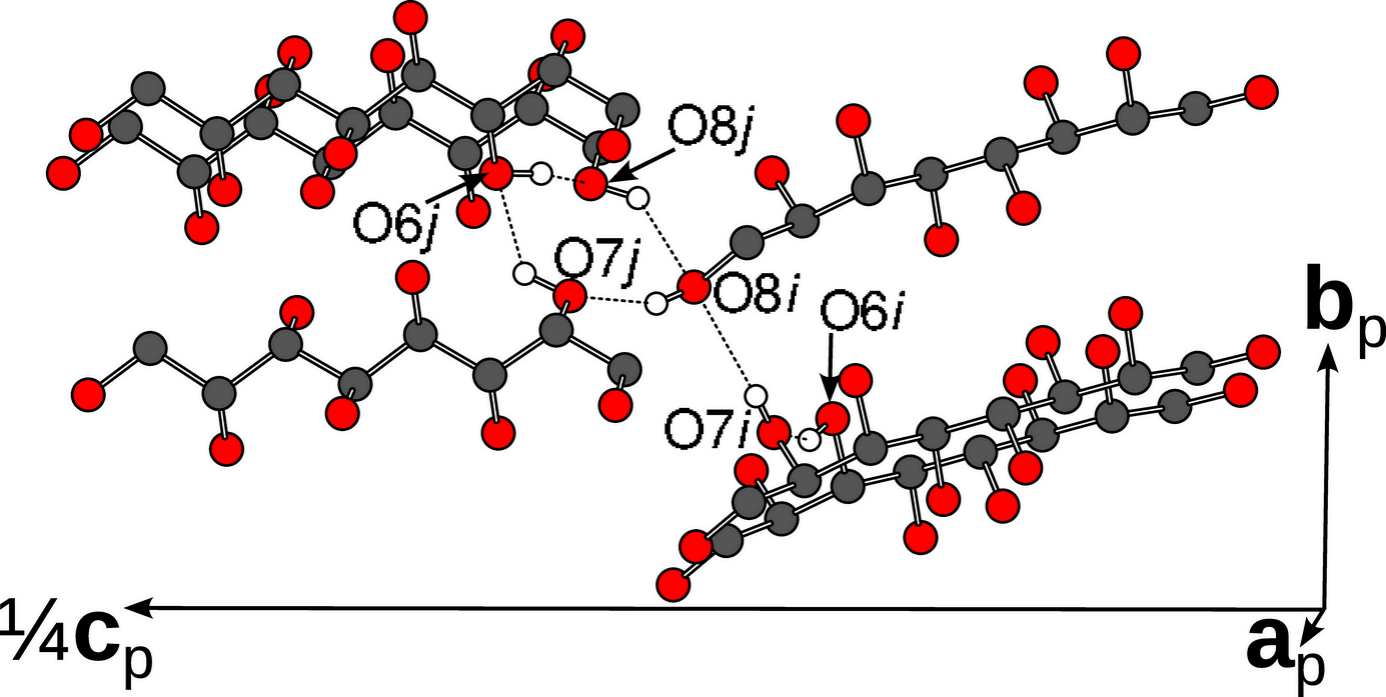
Hydrogen-bonding network in the *d*/*b* layers, exemplified by an *i*/*j* pair of molecules, viewed slightly inclined to [100]. Atoms as given in Fig. 5 ▸.

Then let us turn our attention to the hydrogen bonds in *d*/*b* layers, exemplarily shown in Fig. 7 ▸. Here, only a single network is observed, described by the graph
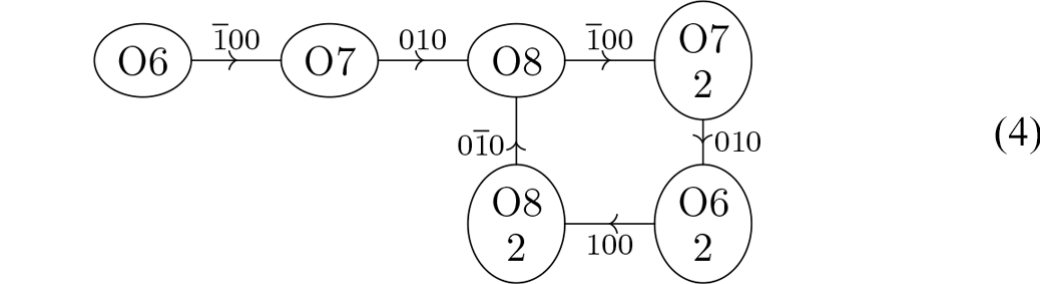
The core of the network is a four-atom cycle where all atoms belong to distinct molecules. Molecules obtained by a twofold rotation in the 〈110〉 direction of the *P*4_3_2_1_2 pseudo-symmetry are marked by ‘2’. This twofold rotation symmetry is broken precisely by this hydrogen-bonding network. Additionally an O6 and an O7 atom form a linear side chain. The hydrogen bond of the O7 atom donating into the cycle is distinctly weaker than the others (O7⋯O8 distance ∼3.1 Å, see Table 2 ▸).

The graph in equation (4) clearly shows that the hydrogen-bonding network is incompatible with the pseudo-twofold rotation symmetry as the O6 and O7 atoms are only part in the cycle for one but not the other molecule. Formally, we can say that the permutation that exchanges the atoms supposedly equivalent by pseudo-symmetry is not an automorphism of the graph and therefore the twofold rotation symmetry cannot be realized.

## 4. Conclusion and outlook

One might assume that a crystal structure with an ≈ 88 Å axis is complex or low in symmetry. **GalC8** is not: according to the *P*4_3_2_1_2 pseudo-symmetry it is built of only one crystallographically equivalent molecule. The long axis is due to the linear arrangement of the molecules. However, symmetry is broken locally by the hydrogen-bonding network, whereby the lower-symmetry part of the structure may appear in two orientations. Thus an infinite number of locally equivalent arrangements may exist, which, if periodic, all possess a *c* axis of a multiple of ≈ 88 Å. **GalC8** does not adopt the simplest of these polytypes, which means that there is some sort of long-range information transfer during crystal growth.

Clearly, a generalization of space group to partial symmetry is required since it reflects the locality of interatomic interactions. We suggest not considering disjoint subsets (such as layers) as being the objects of the corresponding groupoids, but rather MERs that partially overlap. This overlap expresses the local equivalence of the members of structure families.

The structural elucidation of the remaining long-chain sugar alcohols we have synthesized is in progress and we are looking forward to more interesting surprises.

## Supplementary Material

Crystal structure: contains datablock(s) I. DOI: 10.1107/S2052520626004907/ne5017sup1.cif

Structure factors: contains datablock(s) I. DOI: 10.1107/S2052520626004907/ne5017Isup2.hkl

CCDC reference: 2553234

## Figures and Tables

**Table 1:** Data collection and refinement details for **GalC8**

Crystal data	
Chemical formula	C_8_H_18_O_8_
*M*_*r*_	242.22
Crystal system	Monoclinic
Space group	*P*2_1_
Temperature (K)	170
*a*, *b*, *c* (Å)	4.87027 (10), 4.86819 (12), 87.8394 (16)
β (°)	90.3132 (17)
*V* (Å^3^)	2082.59 (8)
*Z*, *Z*′	8, 4
Radiation type	Synchrotron
Wavelength, λ (Å)	0.560
ρ_calc_ (g cm^−3^)	1.545
μ (mm^−1^)	0.084
Crystal shape, color	Plate, colorless
Crystal size (mm)	0.10 × 0.09 × 0.01

Data collection	
Diffractometer	Huber Eulerian cradle
Absorption correction	Multi-scan
*T*_min_, *T*_max_	0.653, 1.000
No. of measured, independent and observed [*I* > 3σ(*I*)] reflections	34215, 10304, 9099
*R*_int_	0.0770
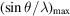 (Å^−1^)	0.812

Refinement	
*R*_obs_	0.1129
*wR*(*F*^2^)_obs_	0.2903
*R*_all_	0.1196
*wR*(*F*^2^)_all_	0.2954
Goodness of fit	1.051
No. of parameters	675
No. of restraints	74
Δρ_min_, Δρ_max_ (e Å^−3^)	−0.51, 0.86
Twin operation	4_[001]_
Twin volume ratio	79:21.0 (5)

**Table 2:** O⋯O distances *d* of hydrogen-bond connected atoms in **GalC8** O⋯H distances are not given, because the positions of the H atoms are not well determined.

Atoms	*d* (Å)	Atoms	*d* (Å)
O1*i*⋯O1*i*	2.824 (6)	O1*k*⋯O1*j*	2.839 (9)
O2*i*⋯O4*i*	2.737 (9)	O2*k*⋯O4*k*	2.724 (7)
O3*i*⋯O2*i*	2.695 (7)	O3*k*⋯O2*k*	2.669 (9)
O4*i*⋯O5*i*	2.677 (8)	O4*k*⋯O5*k*	2.659 (9)
O5*i*⋯O3*i*	2.705 (9)	O5*k*⋯O3*k*	2.696 (7)
O6*i*⋯O7*i*	2.806 (8)	O6*k*⋯O7*k*	2.789 (10)
O7*i*⋯O8*i*	3.138 (12)	O7*k*⋯O8*k*	3.115 (10)
O8*i*⋯O7*j*	2.755 (8)	O8*k*⋯O7*l*	2.759 (9)
O1*j*⋯O1*k*	2.832 (9)	O1*l*⋯O1*l*	2.823 (6)
O2*j*⋯O4*j*	2.744 (7)	O2*l*⋯O4*l*	2.746 (9)
O3*j*⋯O2*j*	2.698 (10)	O3*l*⋯O2*l*	2.695 (7)
O4*j*⋯O5*j*	2.688 (10)	O4*l*⋯O5*l*	2.702 (7)
O5*j*⋯O3*j*	2.730 (7)	O5*l*⋯O3*l*	2.754 (9)
O6*j*⋯O8*j*	2.781 (8)	O6*l*⋯O8*l*	2.769 (10)
O7*j*⋯O6*j*	2.705 (10)	O7*l*⋯O6*l*	2.726 (7)
O8*j*⋯O8*i*	2.963 (11)	O8*l*⋯O8*k*	2.932 (10)
